# A Novel Approach to Lower-limb Axial Alignment Analysis: A CT Study

**DOI:** 10.5435/JAAOSGlobal-D-19-00139

**Published:** 2019-11-27

**Authors:** Maximiliano Barahona, Mauricio Guzman, Cristian Barrientos, Alvaro Zamorano, Miguel Palet, Carlos Infante, Jaime Hinzpeter

**Affiliations:** From the Orthopaedic Department (Dr. Barahona, Dr. Barrientos, Dr. Zamorano, Dr. Palet, Dr. Infante, and Dr. Hinzpeter) and the Radiology Department (Dr. Guzman), Hospital Clinico Universidad de Chile, Santiago, Chile.

## Abstract

To analyze the torsion of the lower extremities in a healthy cohort and to determine the contribution of different segments of the femur and tibia to the torsion of both bones.

**Methods:**

In this cross-sectional study, 32 patients with nonjoint or bone-related symptoms were analyzed by CT angiography. Lower-limb torsion, femoral torsion, proximal femoral torsion, femoral shaft torsion, distal femoral torsion, tibial torsion, proximal tibial torsion, and distal tibial torsion were measured.

**Results:**

The median total limb torsion was 25° external torsion, with the median femoral torsion being −9° and the median tibial torsion 30°. Both femoral metaphyses had internal torsion, with the internal torsion of the proximal metaphysis being approximately three times greater than that of the distal femoral metaphysis. The shaft was found to compensate with an external torsion of approximately two-thirds of the internal torsion of both femoral metaphyses. The proximal metaphysis of the tibia accounted for approximately one-third of the external torsion, with the segment from the distal to the tibial tubercle accounting for the remaining two-thirds of the tibial torsion.

**Conclusions:**

The diaphysis and distal metaphysis are the major contributors to external torsion of the tibia, whereas the proximal metaphysis is the major contributor to the internal torsion of the femur.

Several pathologic conditions of the hips and knees can be affected by axial alignment of the lower limbs.^1^ For example, failure in some patients undergoing revision hip arthroscopy has been associated with excessive internal or external torsion of the femur,^2^ and patellofemoral instability has been associated with torsional problems of the femur and/or tibia.^3^ Excessive internal torsion of the femur may lead to trochlear dysplasia, as illustrated in a proposed model for hip dysplasia.^4^

For the purposes of nomenclature, torsion is the optimal term used to describe axial alignment because torsion refers to the twisting of an object on itself. Rotation is regarded as the optimal term to describe movements of two objects with respect to each other, as during an osteotomy. The use of other terms is not encouraged.^5^

It is unclear in which segment of the femur and tibia should preferably undergo derotational osteotomy (DO), although studies have suggested that the site of DO should depend on the affected joint. For example, patients with hip pain due to excessive internal torsion should undergo DO on the proximal femur.^6^ Other studies have suggested that the segment chosen for osteotomy should depend on coronal alignment; for example, if a femur is in valgus and has excessive internal torsion, the osteotomy should be done on the distal femur.^7^ Alternatively, the choice of segment may be dependent on surgical results; for example, the higher rate of complications after DO on the proximal than on the distal tibia has suggested that DO should be done on the distal tibia.^8^ However, considerations of coronal alignment have suggested that tibial proximal DO may yield better results in patients who have also a varus knee.^8^

These considerations are important, however, when analyzing defects in the coronal plane. Analyses are based on the segment involved, both by measuring the center of angulation (CORA) and by angles of the proximal and distal mechanical axes of the femur and tibia.^9^ It is also essential to analyze the axial alignment of the affected segment of the tibia or femur. The affected segment is obvious in axial malalignment after a fracture in both bones, but this does not apply to other pathologic conditions, such as patellar instability.

The purposes of this study were to analyze the torsion of the lower extremities in a healthy cohort and to determine the contribution of different segments of the femur and tibia to the torsion of both bones.

## Methods

This cross-sectional study involved patients who underwent CT angiography (CTA) of the lower limbs at our institution between 2014 and 2016. The study protocol was approved by the institutional ethics review board at our institution. Participants provided written, informed consent before inclusion. CTA results were reviewed, and patients who showed any signs of arthrosis in the hips, knees, or ankles, or had surgical stigmas after previous surgeries (eg, osteosynthesis material such as plates) were excluded. Patients who met the inclusion and exclusion criteria were contacted by telephone; those contacted successfully were asked the following questions: (1) Have you ever had surgery on your hips, knees, or ankles?, (2) Have you ever consulted an orthopaedic surgeon for hip, knee, or ankle pain?, (3) Have you had hip, knee, or ankle pain in the last 2 years?, and (4) Do you have trouble climbing up or down stairs because of a leg impairment? Patients who answered “no” to all four questions were included.

CTA images were obtained on patients in the supine position, with the knee at full extension, using a Multi-Slice CT scanner (Somaton Sensation 64; Siemens). The field of view was from the celiac trunk to both feet. The technical parameters were Kv 120, 200 mAs; rotation time, 0.33 seconds; 64 × 0.6-mm detector collimation; slice thickness, 2 mm; increment, 1 mm; pitch, 0.45; and a kernel bilateral filter (B30 f). Images were analyzed and measurements done by a single experienced musculoskeletal radiologist using the OsiriX v4.0 program. A negative value was defined as internal torsion and a positive value as external torsion.

**Figure 1:**
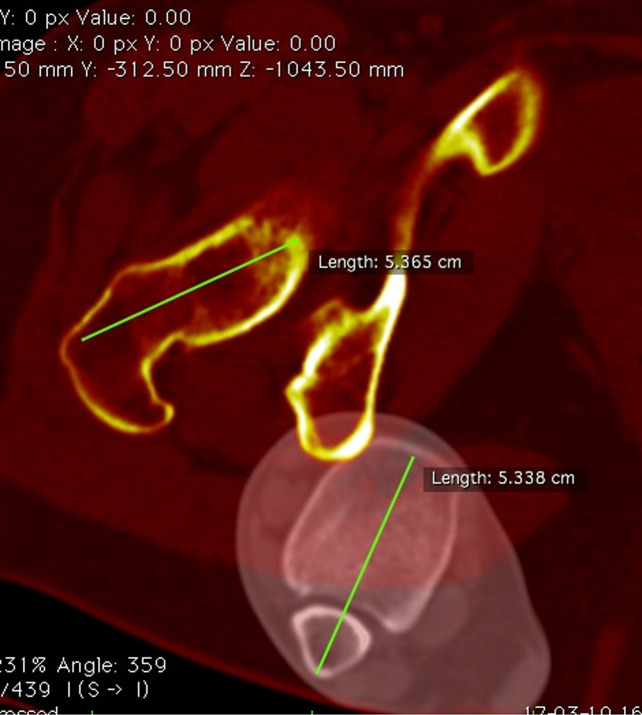
Lower-limb torsion. Superposition of two CT slices. The femoral slice is in the proximal femur, and the tibial slice is in the malleolus. Limb torsion is measured as the angle between a line in the femoral neck axis and a line joining the center of the medial and the lateral malleolus.

All parameters were measured after the superpositioning of two CT cuts. Limb torsion (LT) was measured by the superposition of a femoral CT slice in the proximal femur and a tibial CT slice at the malleolus. The angle between the femoral neck axis and a line joining the center of the medial and the lateral malleolus was measured to determine LT (Figure 1).

**Figure 2:**
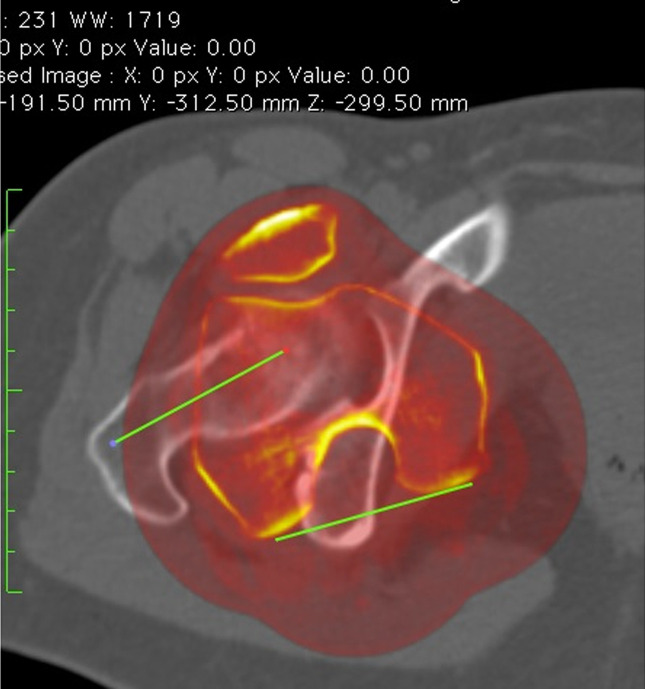
Femoral torsion. Superposition of two CT slices. The first slice is in the proximal femur, and the second slice is in the distal femur. Femoral torsion is measured as the angle between a line in the femoral neck axis and a line joining the posterior borders of both condyles.

Femoral torsion (FT) was measured by the superposition of a CT slice in the proximal femur and a CT slice in the distal femur. The angle between the femoral neck axis and a line joining the posterior border of both condyles was measured to determine FT (Figure 2).

**Figure 3:**
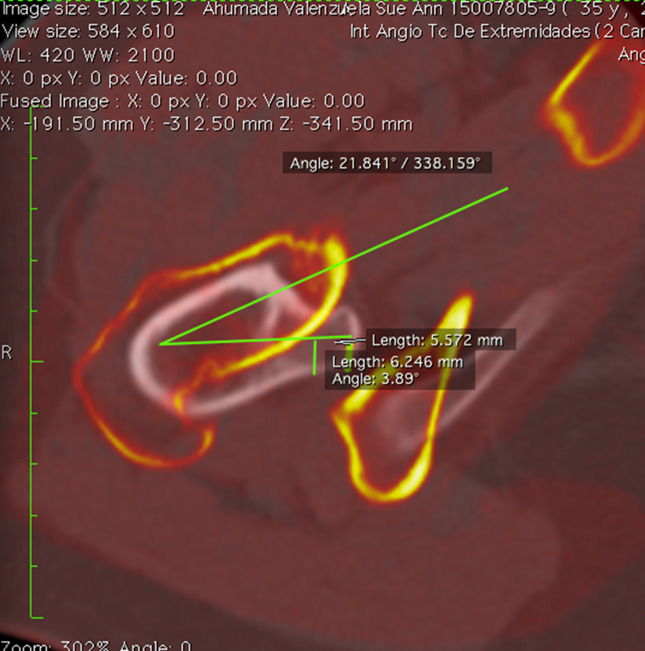
Proximal femoral torsion. Superposition of two CT slices. The first slice is the proximal femur in the tip of the greater trochanter, and the second slice is in the center of the lesser trochanter. Proximal femoral torsion is measured as the angle between a line in the femoral neck axis and a line joining the center of the lesser trochanter.

Proximal femoral torsion was measured by the superposition of a CT slice at the tip of the greater trochanter and a CT slice in the center of the lesser trochanter. The angle between the femoral neck axis and a line joining the center of the lesser trochanter was measured to determine proximal femoral torsion (Figure 3).

**Figure 4:**
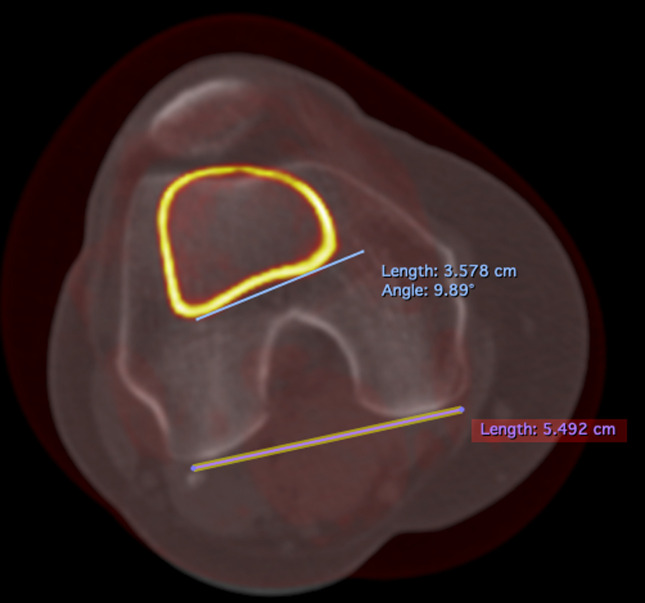
Distal femoral torsion. Superposition of two CT slices. Distal femoral torsion angle is measured as the angle between a line in the posterior cortex at the level of the diaphysis-metaphyseal distal junction and a line joining the posterior borders of both condyles.

Distal femoral torsion was measured by the superposition of a CT slice at the level of the diaphysis-metaphyseal distal junction and a CT slice in the distal femur. The femoral diaphyseal-distal metaphysis junction was determined by measuring the width of the femoral side in a coronal view of the knee joint line; this distance was projected proximally, with the slice at which the line ends selected as the union between the diaphysis and the distal metaphysis. The angle between a line in the posterior cortex at the level of the diaphysis-metaphyseal distal junction and a line that joins the posterior border of both condyles was measured to determine distal femoral torsion (Figure 4).

Femoral shaft torsion was measured by the superposition of a CT slice at the level of the center of the lesser trochanter and a CT slice at the level of the diaphysis-metaphyseal distal junction. The angle between a line in the center of the lesser trochanter and line in the posterior cortex at the level of the diaphysis-metaphyseal distal junction was measured to determine femoral shaft torsion.

**Figure 5:**
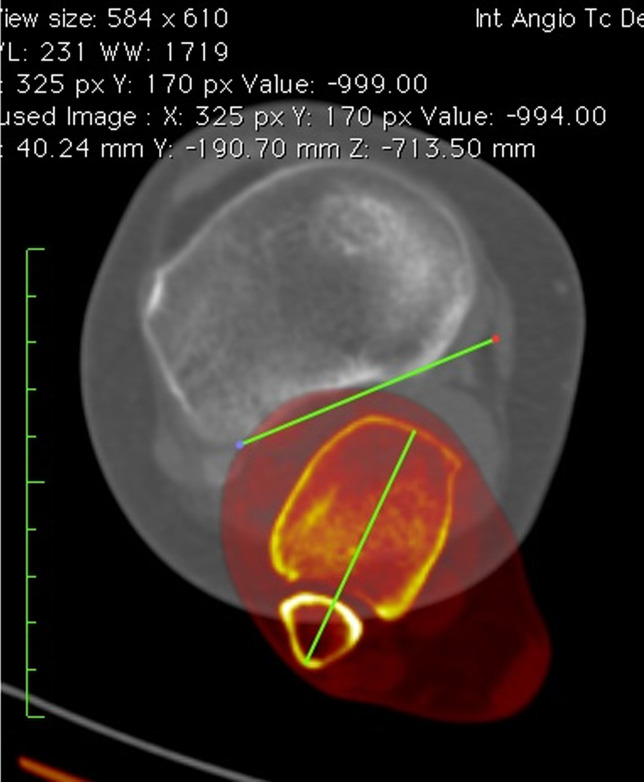
Tibial torsion. Superposition of two CT slices. The first slice is in the proximal tibia, and the second slice is in the distal tibia. Tibial torsion is measured as the angle between a line joining the posterior borders of the tibial plateaus at the level of the tibial insertion of the posterior cruciate ligament and a line joining the center of the medial and the lateral malleolus.

Tibial torsion (TT) was measured by the superposition of a CT slice in the proximal tibia and a CT slice in the distal tibia. The angle between a line joining the posterior border of the tibial plateau at the level of the tibial insertion of the posterior cruciate ligament (PCL) and a line joining the center of the medial and the lateral malleolus was measured to determine TT (Figure 5).

**Figure 6:**
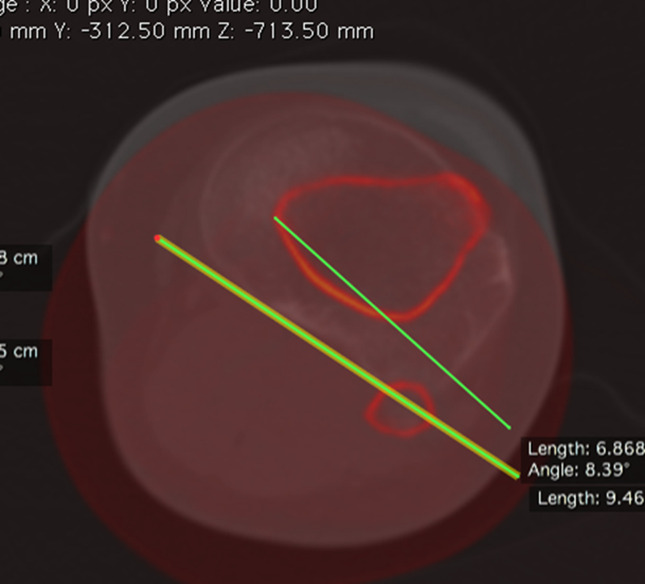
Proximal tibial torsion. Superposition of two CT slices. The first slice is in the proximal tibia, and the second slice is at the level of the tibial tubercle. Proximal tibial torsion is measured as the angle between a line joining the posterior border of the tibial plateaus at the level of the tibial insertion of the posterior cruciate ligament and a line joining the posterior border of the tibia at the level of the tibial tubercle.

Proximal tibial torsion was measured by the superposition of a CT slice in the proximal tibia and a CT slice at the level of the tibial tubercle. The angle between a line joining the posterior border of the tibial plateau at the level of the tibial insertion of the PCL and a line joining the posterior border of the tibia at the level of the tibial tubercle was measured to determine proximal tibial torsion (Figure 6).

**Figure 7:**
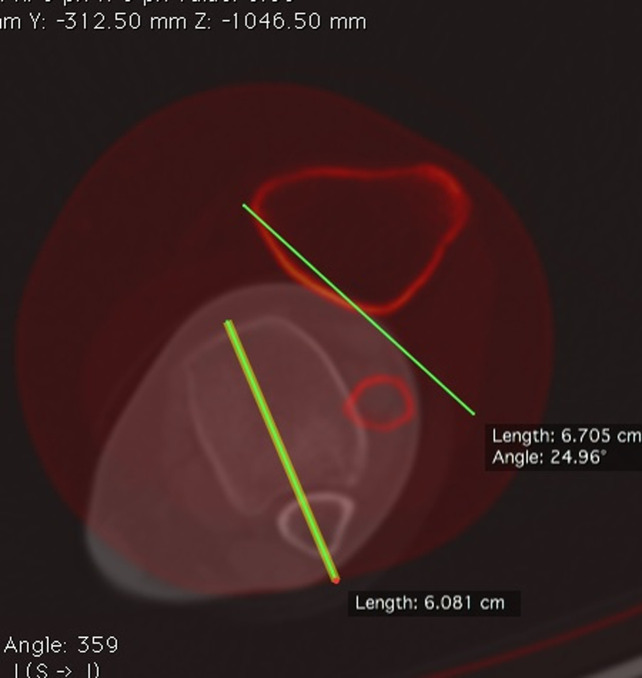
Distal tibial torsion. Superposition of two CT slices. The first slice is at the level of the tibial tubercle, and the second slice is at the malleolus. Distal tibial torsion is measured as the angle between a line joining the posterior border of the tibia at the level of the tibial tubercle and a line joining the center of the medial and the lateral malleolus.

Distal tibial torsion was measured by the superposition of a CT slice at the level of the tibial tubercle and a CT slice at the malleolus. The angle between a line joining the posterior border of the tibia at the level of the tibial tubercle and a line joining the center of the medial and lateral malleolus was measured to determine distal tibial torsion (Figure 7).

Knee torsion was measured by the superposition of a CT slice in the distal metaphysis and a CT slice at the level of insertion of the PCL in the tibia. The angle between a line joining both posterior femoral condyles and a line joining the posterior cortex of the tibial plateaus at the level of the PCL insertion was measured to determine DT.

For statistical analysis, results were reported as the median and interquartile range. All variables were analyzed by the Shapiro-Wilk test to determine whether they were distributed normally, with normality defined as a probability >0.15. All statistical analyses were done using STATA v.11.1 software.

## Results

**Figure 8:**
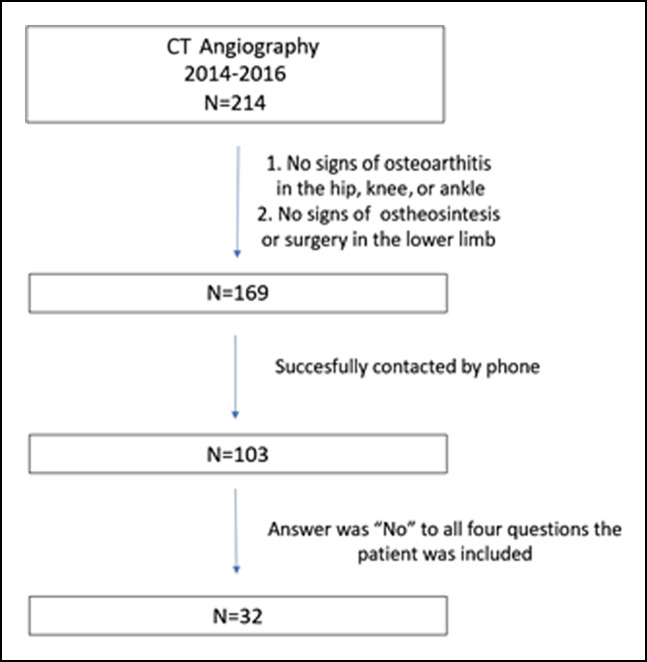
Patient flowchart.

A review of medical records at our institution identified 214 patients who underwent CTA. Of these, 169 met the inclusion and exclusion criteria. Attempts were made to contact these patients by telephone, with 103 patients contacted successfully. Thirty-two patients were included in the study; a study flowchart is shown in Figure 8. The median age of these 32 patients was 55 years (interquartile range, 48 to 66 years), and 18 (56%) were men.

The median total LT was 25° external torsion, with the median FT being −9° and the median tibial torsion 30° (Table 1).

**Table 1:** Parameters Measured in the 32 Patients Included in This Study

Measurement	Right	Left	Total
LT	25° (20° to 30°)	25° (17° to 29°)	25° (19° to 30°)
FT	−9° (−5° to −14°)	−10° (−5°to −14°)	−9° (−5° to −14°)
pTT	−24° (−18° to −32°)	−23° (−18° to −30°)	−24° (−18° to −32°)
Shaft femoral torsion	21° (18° to 30°)	23° (18° to 26°)	21° (18° to 28°)
dFT	−8 (−6° to −11°)	−8° (−5° to −11°)	−8 (−6° to −11°)
TT	30° (25° to 34°)	30° (23° to 33°)	30° (24° to 34°)
pTT	9° (6° to 13°)	8° (4° to 10°)	9° (5° to 12°)
dTT	19° (12° to 27°)	21° (12° to 26°)	20° (12° to 26°)
KT	5° (2° to 7°)	5° (2° to 11°)	5° (2° to 8°)

dFT = distal femoral torsion, dTT = distal tibial torsion, FT = femoral torsion, LT = limb torsion, pFT = proximal femoral torsion, TT = tibial torsion

All results are reported as median (interquartile range).

**Figure 9:**
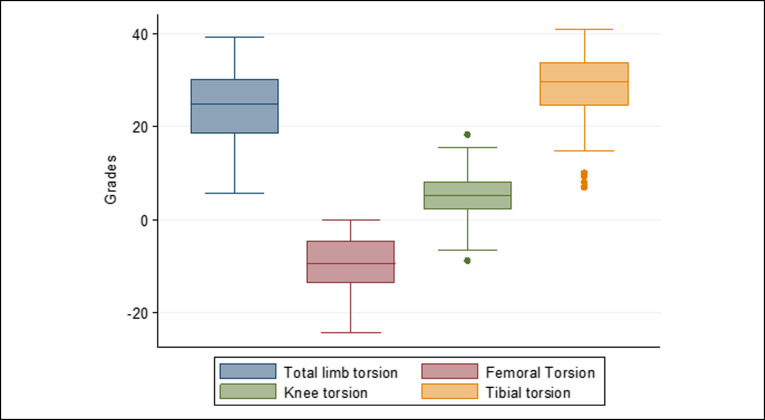
Distribution of limb torsion, femoral torsion, tibial torsion, and articular torsion. Only femoral torsion contributed to internal torsion. The global alignment was in external torsion.

**Figure 10:**
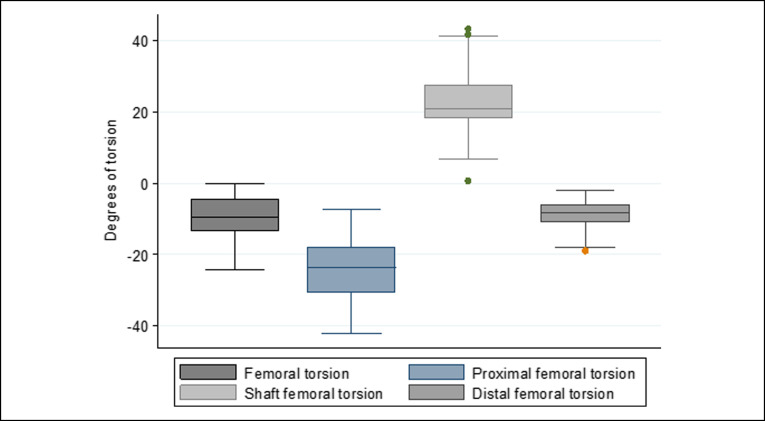
Distribution of femoral torsion. Both metaphyses had internal torsion, with the shaft having external torsion. Internal torsion was about three times higher for the proximal for the distal metaphysis. The shaft compensated for approximately two-thirds of the internal torsion of both metaphyses.

**Figure 11:**
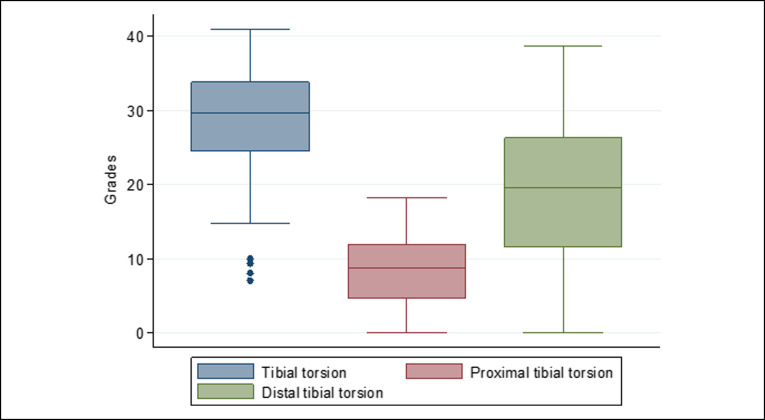
Distribution of tibial torsion. Both segments had external torsion. Proximal torsion was approximately one-third of tibial torsion.

Both femoral metaphyses had internal torsion, with the internal torsion of the proximal metaphysis being approximately three times greater than the internal torsion of the distal femoral metaphysis. The shaft was found to compensate with an external torsion of approximately two-thirds of the internal torsion of both femoral metaphyses. The proximal metaphysis of the tibia accounted for approximately one-third of the external torsion, with the segment from the distal to tibial tubercle accounting for the remaining two-thirds of the tibial torsion. The distributions of limb, femoral, and tibial torsion are shown in Figures 9–11, respectively.

## Discussion

Lower-limb alignment in the frontal plane is well documented, and osteotomy planning has been validated. Initially, the mechanical axis is drawn in the coronal plane. If valgus or varus alignment is diagnosed, both the distal and proximal angles of the femur and tibia are measured to determine the specific segment to do the osteotomy. Also, if the deformities are distant from the knee, CORA is measured.^10,11^ The same principles are used in the sagittal plane, with special attention paid to the tibial slope, as osteotomy is also done to correct these defects.^12^

For axial alignment, the analysis has not yet been standardized. Normal values for the femur depend on the anatomical landmarks used to measure torsion, with differences of up to 10° reported.^13^ The anatomical landmarks used to measure torsion should be chosen by consensus of the orthopaedic surgeon and the radiologist.^5^

The relationship between pathologic findings and axial malalignment must also be determined. For example, femoroacetabular impingement may be associated with excess internal torsion of the femoral neck, but the threshold for doing an osteotomy rather than a hip arthroscopy has not been documented. This problem is also applicable to patellofemoral pathologies, with the medial patellofemoral ligament, the distance between the tibial tubercle and trochlear groove, and trochlear dysplasia regarded as more relevant than axial alignment.

The determination of the site of osteotomy in patients with torsion malalignment is not as in patients with coronal malalignment. It may not be possible to determine CORA for axial alignment because differences among segments of the femur and tibia are likely to be minimal, except in patients with malunion after a fracture.

The main contribution of this study was to describe torsion in different segments of the tibia and femur. Measurements of torsion in individual segments may be a tool to determine the segment at which osteotomy can be best done. Analysis of individual segments is required if any alterations in the lower-limb, femoral, and tibial torsion are observed. The femur can be divided into three segments because three different sites in the femur are proposed for DO: proximal femur metaphysis,^14^ diaphysis (subtrochanteric and distal diaphysis),^1^ and distal metaphysic.^15^ The tibia can be divided into two segments because two different sites for DO in the tibia are proposed: proximal metaphysis and distal metaphysic.^8^

This study had several limitations. First, all measurements were done by a single radiologist; therefore, interobserver bias was not determined. However, this radiologist specializes in musculoskeletal radiology and does all lower-limb CT scan for torsional analysis in our institution. Also, previous studies reported excellent inter-rater reliability, suggesting that bias was minimal. These parameters are routinely measured in patients at our institution with suspected torsional malalignment and in patients with patellar instability.

Although the sample size was underpowered to estimate normal values for the general cohort, it can be used as a guide to determine the segment for DO of the femur or tibia. Comparing the axial alignment of any patient to the results of this study will allow to identify the specific segment of the bone that is contributing the most to the mal torsion. For example, if the internal femur torsion is increased, but the proximal segment is increased only 10%, and the external torsion of the femoral diaphysis is decreased 50%, then the diaphysis probably should be addressed. This strategy has been proposed for patients with patellar instability and excessive internal FT but has not yet been used for all patients with torsional-related problems in the femur and tibia.^16^ Validation of this approach to axial malalignment requires determination of patient outcomes after surgery.

## Conclusions

The diaphysis and distal metaphysis are the major contributors to external torsion of the tibia, whereas the proximal metaphysis is the major contributor to internal torsion of the femur. Systematic analysis of axial alignment may help determine the most appropriate segment of the bone to do a DO.

## References

[1] Dickschas J Harrer J Reuter B Schwitulla J Strecker W: Torsional osteotomies of the femur. J Orthop Res 2015;33:318-324. 25399673 10.1002/jor.22758

[2] Ricciardi BF Fields K Kelly BT Ranawat AS Coleman SH Sink EL: Causes and risk factors for revision hip preservation surgery. Am J Sports Med 2014;42:2627-2633. 25139303 10.1177/0363546514545855

[3] Teitge RA: Patellofemoral syndrome a paradigm for current surgical strategies. Orthop Clin North Am 2008;39:287-311. 18602559 10.1016/j.ocl.2008.04.002

[4] Liebensteiner MC Ressler J Seitlinger G Djurdjevic T El Attal R Ferlic PW: High femoral anteversion is related to femoral trochlea dysplasia. Arthroscopy 2016;32:2295-2299. 27209622 10.1016/j.arthro.2016.03.023

[5] Schröter S Nakayama H Ihle C: Torsional osteotomy. J Knee Surg 2019; Feb 8 [Epub ahead of print]. 10.1055/s-0039-1678677 30736056

[6] Schröter S Elson DW Ateschrang A: Lower limb deformity analysis and the planning of an osteotomy. J Knee Surg 2017;30:393-408. 28599326 10.1055/s-0037-1603503

[7] Nelitz M Wehner T Steiner M Dürselen L Lippacher S: The effects of femoral external derotational osteotomy on frontal plane alignment. Knee Surg Sports Traumatol Arthrosc 2014;22:2740-2746. 23887859 10.1007/s00167-013-2618-5

[8] Krengel WF III Staheli LT: Tibial rotational osteotomy for idiopathic torsion. A comparison of the proximal and distal osteotomy levels. Clin Orthop Relat Res 1992;283:285-289. 1395261

[9] Smith JO Wilson AJ Thomas NP: Osteotomy around the knee: Evolution, principles and results. Knee Surg Sports Traumatol Arthrosc 2013;21:3-22. 23052110 10.1007/s00167-012-2206-0

[10] Lobenhoffer P Van Heerwaarden RJ Staubli AE: Osteotomies Around the Knee: Indications-Planning-Surgical Techniques Using Plate Fixators. Stuttgart, Thieme, 2011.

[11] Paley D: Principles of Deformity Correction. Berlin and Heidelberg, Springer, 2014.

[12] Dejour D Saffarini M Demey G Baverel L: Tibial slope correction combined with second revision ACL produces good knee stability and prevents graft rupture. Knee Surg Sports Traumatol Arthrosc 2015;23:2846-2852. 26298711 10.1007/s00167-015-3758-6

[13] Kaiser P Attal R Kammerer M: Significant differences in femoral torsion values depending on the CT measurement technique. Arch Orthop Trauma Surg 2016;136:1259-1264. 27501703 10.1007/s00402-016-2536-3 PMC4990621

[14] Kamath AF Ganz R Zhang H Grappiolo G Leunig M: Subtrochanteric osteotomy for femoral mal-torsion through a surgical dislocation approach. J Hip Preserv Surg 2015;2:65-79. 27011816 10.1093/jhps/hnv011 PMC4718471

[15] Imhoff FB Beitzel K Zakko P: Derotational osteotomy of the distal femur for the treatment of patellofemoral instability simultaneously leads to the correction of frontal alignment: A laboratory cadaveric study. Orthop J Sports Med 2018;6:2325967118775664-. 29900182 10.1177/2325967118775664 PMC5985607

[16] Seitlinger G Moroder P Scheurecker G Hofmann S Grelsamer RP: The contribution of different femur segments to overall femoral torsion. Am J Sports Med 2016;44:1796-1800. 27159300 10.1177/0363546516639945

